# Heart rate variability response to low-frequency sounds vibrations in regularly active male subjects

**DOI:** 10.3389/fspor.2025.1573660

**Published:** 2025-06-27

**Authors:** Rafaël Hauser, Cyril Besson, Francis Degache, Vincent Gremeaux

**Affiliations:** ^1^ Faculty of Biology and Medicine, University of Lausanne, Lausanne, Switzerland; ^2^ Sports and Exercise Medicine Center, Swiss Olympic Medical Center, Lausanne University Hospital, Lausanne, Switzerland; ^3^ Institute of Sports Sciences, University of Lausanne, Lausanne, Switzerland; ^4^ Sports Sciences Department, AudioVitality, Lausanne, Switzerland

**Keywords:** heart rate variability, autonomic nervous system, whole-body vibration, low-frequency sound, recovery, sport performance

## Abstract

**Objective:**

Low-Frequency Vibration (LFV) is a type of sound therapy used for relaxation and stress management. This study investigated the effects of LFV on heart rate variability (HRV), compared to a session without any vibrations (No-vibration) in healthy male participants.

**Methods:**

Intra-individual comparative study: participants experienced two blinded 40-minutes sessions, separated by a week of wash-out period, a LFV and a No-vibration one, in a soundproof environment. HRV temporal and frequential parameters were measured before, during, and after each session.

**Results:**

Both sessions showed a decrease in heart rate between pre-session (64.2 ± 1.9 and 61 ± 1.9 BPM) and during intervention (58.7 ± 2.1 and 58.6 ± 1.7). Only LFV was associated with enhanced HRV variables at 30 min post-intervention compared to pre-session (78.9 ± 15.1 u.a vs. 112.6 ± 27.8 u.a). LFV significantly increased parasympathetic activity, as evidenced by higher HRV variables measures 30 min post-session, compared to the No-vibration session (*p* = 0.007).

**Conclusion:**

Vagal tone was improved 30 min after a LFV session in healthy active male participants, indicating its potential utility as a recovery modality. Further research is warranted to assess long-term effects and applications in diverse populations.

## 1 Introduction

Vibroacoustic therapy (VAT) is a type of sound therapy that involves passing low frequency sine-wave vibrations into the body. This method aims to provide both auditory and vibratory stimuli (1). Many recent studies have analyzed the effects of VAT on pain management and on spasticity in post-stroke neurorehabilitation showing a neuromodulator and a muscle relaxation effect (2, 3). Low-frequency vibration (LFV) is a form of VAT that is emphasizes its effect on vibrations and is not sound-based. However there is significant variability in how VAT is applied across studies, including differences in frequency ranges, session durations, and exposure conditions (4). In most research, a frequency of 40 Hz is used for its potential relaxation effects after a 20 min exposure (5–7). VAT is also hypothesized to enhance cardiac vagal regulation and physical relaxation by reducing heart rate (HR) (8, 9). Indeed, it has been showed that VAT can interact with tissues through mechanotransduction via the Pacini corpuscles and Merkel cells, directly influencing the autonomic nervous system (ANS) (10). AudioVitality®, a company specialized in VAT, has developed a unique approach involving 40-minute sessions with a combination of 40–80 Hz fundamental frequencies and harmonics. Such harmonic distortions have been shown to generate specific electrical responses in the human brain (11). AudioVitality® RubesaSounds™ apply harmonic distortions in the original and unique context of continuous sounds.

Heart rate variability (HRV), a non-invasive measure of autonomic cardiac control, is increasingly used to assess stress and recovery (12–14). The utility of HRV as a broad-spectrum health indicator with possible application to both clinical and to healthy population has only begun to be explored recently. HRV is offering insight into the balance and interactions between the sympathetic and parasympathetic branches of the ANS (15). High HRV temporal metrics generally indicating greater parasympathetic activity and better recovery (16, 17). Some key HRV metrics, including the root mean square of successive beat-to-beat interval differences (RMSSD) and the high-frequency (HF) power spectrum, have been shown to reflect cardiac vagal tone, giving clinical insight on recovery status in patient (17, 18). Recent studies have shown that VAT, can positively affect the parasympathetic nervous system, particularly in post-exercise recovery (19, 20). Enhanced vagal tone, which correlates with improved well-being and performance, has been observed with multiple interventions using vibrations (21–23). Despite VAT and LFV showing promising benefits in managing stress, the number of objective research on LFV's impact on heart rate variability (HRV) is limited.

The objective of this study was to examine the effects of Low-Frequency Vibration (LFV) on short-term cardiac autonomic regulation in healthy male participants.

## 2 Materials and methods

### 2.1 Study group

Twenty-nine male participants were included in the study. Participants were recruited through posters posted on the university campus, at the sports medicine unit from Lausanne University Hospital or by direct contact with sports club. The participants had to be regularly active [Tier 1–2 of participant Classification Framework (24)], men, between the age of 18 and 40 and in good general health. The participants were excluded from the study if they already participated in a AudioVitality® or any other LFV session, suffered from any acute injury or pathology that may prevent the proper course of the study or endanger their own health. We also excluded participants with known sound hypersensibility and those not accepting to be informed of incidental findings. Every participant was contacted by one of the co-investigators for an initial screening of the inclusion and exclusion criteria. Participants signed an informed consent before the beginning of the study.

In total, 29 participants were included in the study. Among them, two were excluded from the analysis: one because he freely decided to stop after the first session and one who showed signs of arrhythmia on heart rate recordings and was referred for a cardiac check-up. A final number of 27 men were included in the analysis. The mean age of the population was 28 ± 5 years, mean height was 182.8 ± 6.7 cm, and mean weight was 79.4 ± 9.8 kg.

### 2.2 Experimental design

This prospective observational study focused on the state of HRV variables before, during and after a LFV session and a No-vibration session with a within-subjects analysis.

The participants only received partial information on LFV prior to the study to avoid any expectation bias and offer a semi-blinded design. Randomization in the order of the sessions was not achievable to avoid expectation bias because LFV is felts significantly by the participants. All participants went then through the No-vibration session first, and the LFV session second. The participants were told they would go through two different kinds of LFV sessions without any explanations of the No-vibration session. A detailed explanation of the entire process, including a description of both sessions and their differences was given to every participant at the end of the study.

**Figure 1:**
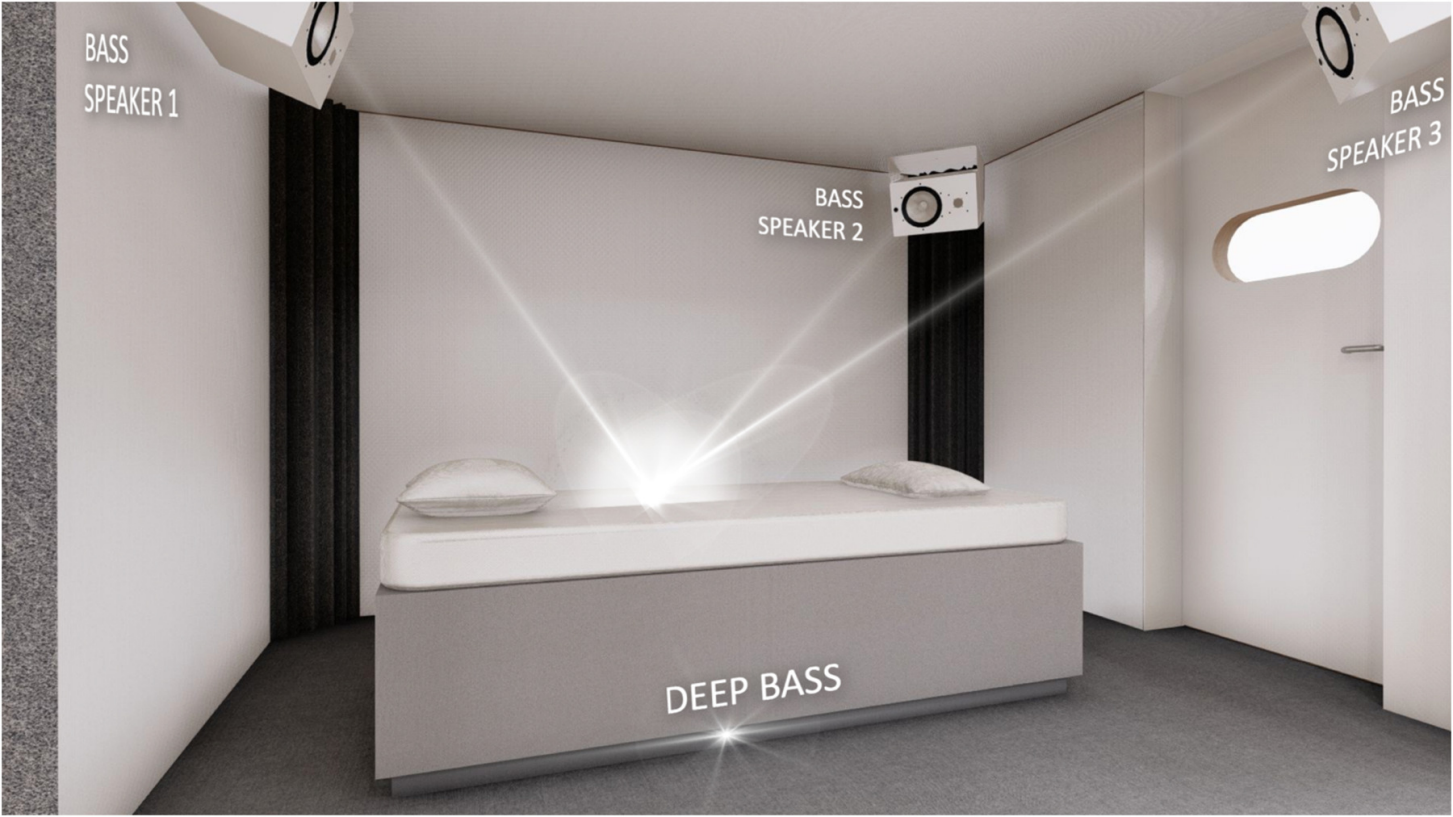
AudioVitality® soundproof studio. Participants were lying on the bed during the entire intervention. Low-frequency sounds vibrations were emitted from the 3 bass speakers, all oriented towards the participant.

Every session took place in AudioVitality® soundproof studios (Figure 1). The participants were lying on a bed for the whole intervention. The beginning of each AudioVitality® session is guided by a pre-recorded voice to help the participant to deeply relax before the session. This 4-minutes introduction was performed in both sessions (No-vibration and LFV). The first session consisted of a 40-minute-long silence with no frequencies emitted (referred as No-vibration in the article). HRV was monitored during the entire session. After the session, the participants stayed in the studios for another 30 min to allow for multiple post-session short-term HRV measurements. The second session took place in the same conditions and consisted of a standardized 40-minute low-frequency sound session. The experimental procedures were explained to all the participants, following the Declaration of 92 Helsinki, and approved by the local ethics committee (CER-VD #2023-01296).

### 2.3 Heart rate variability

#### 2.3.1 Acquisition

R-R intervals were collected during the entire protocol using a heart rate monitor (H10, Polar Electro Oy, Kempele, Finland) (25) and a dedicated smartphone-based application (Polar Sensor Logger app, Jukka Happonen, Finland). The measurement started 5 min before the session and lasted until 30 min post-session.

Analyses were performed in supine position on six different five-minutes samples taken at specific time point. Pre-session (pre), at 20 min during the session (during-20), at 40 min during the session (during-40), 5 min post session (post), 15 min post-session (post-15) and 30 min post-session (post-30).

#### 2.3.2 Confounding factors

As HRV is influenced by many external factors and has high inter-individual variations, a within-subject design has been highly recommended in the literature (26). This design helps to offer optimal experimental control, contribute to the reduction of individual differences in respiratory rates, offer an increased statistical power, and reduce the impact of external factors (17). As testing occurred with 1 week in between, the time of experimentation was kept exactly the same for each individuals to avoid circadian differences in HRV (27).

We designed the study to control as much as possible external factors, including the measuring environment, which was kept as stable as possible (place, time, room temperature, air humidity, noise, devices used, persons present during the measurements). Furthermore, all electronic devices were removed from the proximity of the participant during the study.

We instructed participants to strictly control internal factors, which mainly comprise: stopping intense physical efforts 48 h before examination, stop alcohol and analgesics 12 h before, avoid wearing confining clothes, corsets or supports stockings on the day of their venue and avoid any intake of nicotine, coffee and food 3 h before examination (28). The importance of coming to both visits in similar settings was repeated to the participants.

Prior to the start of the session, participants were asked to go to the bathroom if needed and then lie down in the studio comfortably. To keep the measurements as standardized as possible, communication between the investigator and the participant was restricted to the minimum.

#### 2.3.3 Analyses

The analysis of raw R-R intervals at the 6 time points was conducted using Kubios Premium software (Kubios, Finland). Each dataset underwent a manual and automatic review in Kubios Premium to identify and correct any artifacts or ectopic beats (29, 30). Time- and frequency-domain HRV analyses were conducted on the final 4 min of five-minute R-R interval samples in a supine position, adhering to the international standards (31, 32). Following the latest guidelines by Laborde et al. (17), HRV was assessed in three conditions: resting (measured during a five-minute supine rest prior to sessions), reactivity HRV (analyzing changes in HRV variables from baseline to during-event), and recovery HRV (evaluating changes in HRV variables from during-event to post-event measurements). This three phase experimental structure is advocated as optimal for HRV analysis (17, 33), facilitating comprehensive assessments at each phase and measuring HRV's stimulus-response capabilities. Focus was given to vagally-related HRV variables, logarithmic transformation of the Root Mean Square of Successive Differences (LnRMSSD), Heart Rate (HR) and Low-Frequency + High-Frequency divided by Heart Rate [(LF + HF)/HR]. All the variables are further described in the cited sources (34). We calculated LnRMSSD with a logarithm transformation from RMSSD creating a new variable. Applying a logarithmic transformation (Ln) to RMSSD helps normalize the data distribution, thereby reducing the impact of extreme values and improving comparability between individuals (35). Moreover, LnRMSSD has been shown to be a reliable and sensitive indicator of recovery and training load, particularly in elite athletes (36).

### 2.4 Low-frequency sounds

To accurately analyze inter-participants responses, the exact same protocol of low-frequency sounds was used for every LFVsession. It consisted of twelve, equally long, continuous sounds. Each sound followed the same cycle: 3 s of linear fade-in (from silence to “full intensity”), 3 min of sounds at full intensity (70 dB), 3 s of linear fade-out (from full intensity to silence), and finally 9 s of silence before starting the cycle again for the next sound. At “full intensity”, the sound pressure level, measured at the head was, on average, 72 dB. Each sound contains a low-frequency fundamental frequency (between 40 and 80 Hz) with several of its harmonics. The fundamental frequency was the most prominent (intensity-wise). Harmonics did not exceed 1,000 Hz. The relationship between the sounds' harmonics is a copyrighted technology AudioVitality® RubesaSounds™.

Sounds were diffused through three speakers mounted to the ceiling of the room. All speakers were focused on the center of the room, where the bed was positioned. One speaker was located above the head, and the other two towards the bottom of the bed, one bye each foot. A subwoofer was also used under the bed, pointing towards the bottom of the bed.

### 2.5 Statistical analysis

All data passed normality Shapiro–Wilk test and are expressed as mean ± standard deviation (SD). All data were presented as absolute and as normalized to a percentage of individual pre- values. HRV time-course analysis as a function of session (No-vibration or LFV) was analyzed using a 2 (session) × 6 (recovery time points) simple repeated-measures ANOVA. The Bonferroni correction *post hoc* test was applied when F was significant in the ANOVA. For all statistical analyses, an *α* value of 0.05 was accepted as the level of statistical significance. Statistical analyses were performed with JASP version 0.18.3.

A two-way repeated measures ANOVA with SESSION (LFV vs. No-vibration) and TIME (PRE, DURING, POST) as within-subject factors was conducted to assess the effects of the session type and measurement timing on key HRV-related variables.

In our study, all missing values were handled using the Mean Substitution method. Specifically, we replaced missing values (*n* = 12) with the mean of the available data for the corresponding variable. This method is widely used in scientific research as it helps maintain the overall distribution characteristics of the dataset while minimizing data loss (37). Additionally, mean substitution provides a straightforward approach to handling missing data, especially when the proportion of missing values is low, ensuring that the integrity of the dataset is preserved for subsequent analyses (38).

## 3 Results

### 3.1 Comparison between sessions in pre-session values

**Figure 2:**
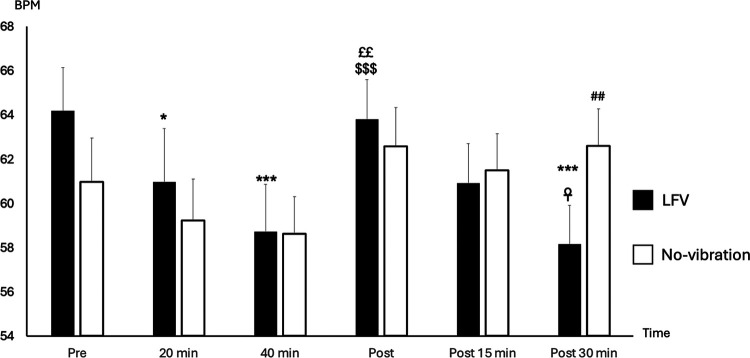
State of HR (BPM) according to time in No-vibration and LFV. State of heart rate (BPM during sessions phases in two tests conditions (black: LFV; white: No-vibration). *p < 0.05 significant differences with Pre; ***p < 0.001 significant differences with Pre; ^££^*p* < 0.01 significant difference with 20 min; ^$$$^*p* < 0.001 significant difference with 40 min; ^☥^*p* < 0.05 significant difference with Post; ^##^*p* < 0.01 significant difference with LFV session. The analysis revealed significant main effects of SESSION [*F*_(1,130)_ = 6.12, *p* = .0147, *η*^2^ ≈ 0.045] and TIME [*F*_(2,130)_ = 5.71, *p* = .0042, *η*^2^ ≈ 0.042]. A highly significant SESSION × TIME interaction was also observed [*F*_(2,130)_ = 36.45, *p* < .0001, *η*^2^ ≈ 0.22], showing a more pronounced heart rate reduction following the LFV session.

**Figure 3:**
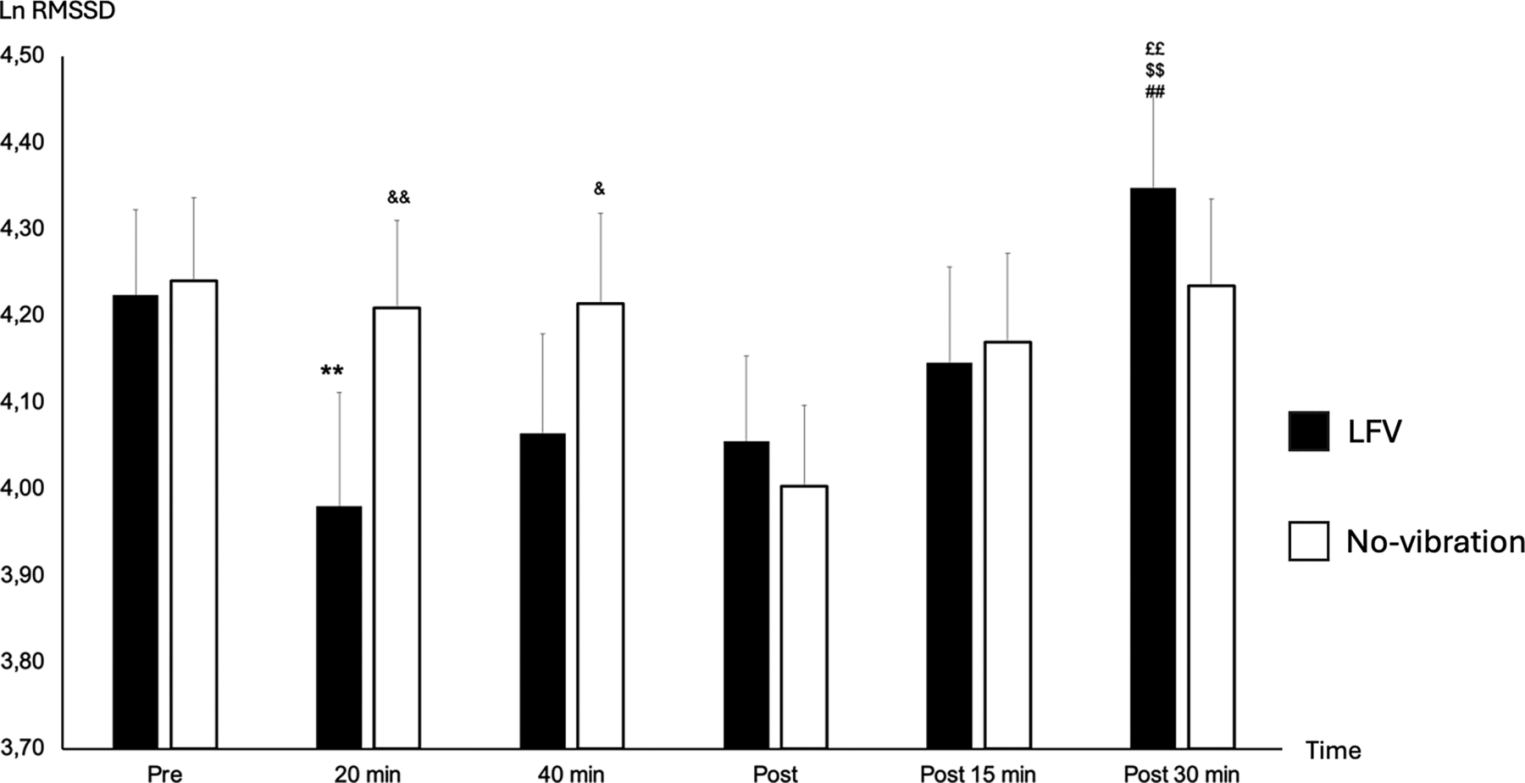
State of lnRMSSD according to time in No-vibration and LFV. State of Ln RMSSD during sessions phases in two tests conditions (black: LFV; white: No-vibration). **p < 0.01 significant differences with PRE; ^££^*p* < 0.01 significant differences with 20 min; ^$$^*p* < 0.01 significant differences with 40 min; ^##^*p* < 0.01 significant differences with post 5 min; ^&^*p* < 0.05 significant differences with LFV session; ^&&^*p* < 0.01 significant differences with LFV session. A significant main effect of SESSION was found [*F*_(1,130)_ = 4.61, *p* = .034, *η*^2^ ≈ 0.035], along with a significant main effect of TIME [*F*_(2,130)_ = 6.56, *p* = .0019, *η*^2^ ≈ 0.048]. There was also a significant SESSION × TIME interaction [*F*_(2,130)_ = 32.51, *p* < .0001, *η*^2^ ≈ 0.20], indicating a stronger time-dependent modulation under the LFV condition.

Pre-session HR was statistically slightly higher in LFV in comparison to No-vibration (*p*-value 0.023) (Figure 2). However, no significant difference was found in pre-session lnRMSSD or (LF + HF)/HR between the two sessions (*p*-value 0.164 and *p*-value 0.645 respectively) (Figure 3).

### 3.2 Reactivity HRV

The reactivity HRVs (between pre and during-20) were not different in all variables for LFV and No-vibration session (*p*-value >0.05). A significant drop in HR between pre- and during-40 was found in both sessions (*p*-value <0.001). Additionally, we found a significant difference between No-vibration and LFV at the during-20 time point showing a drop of RMSSD only in the LFV session (*p*-value <0.001).

### 3.3 Comparison between pre-session and post-30 min in both sessions

**Figure 4:**
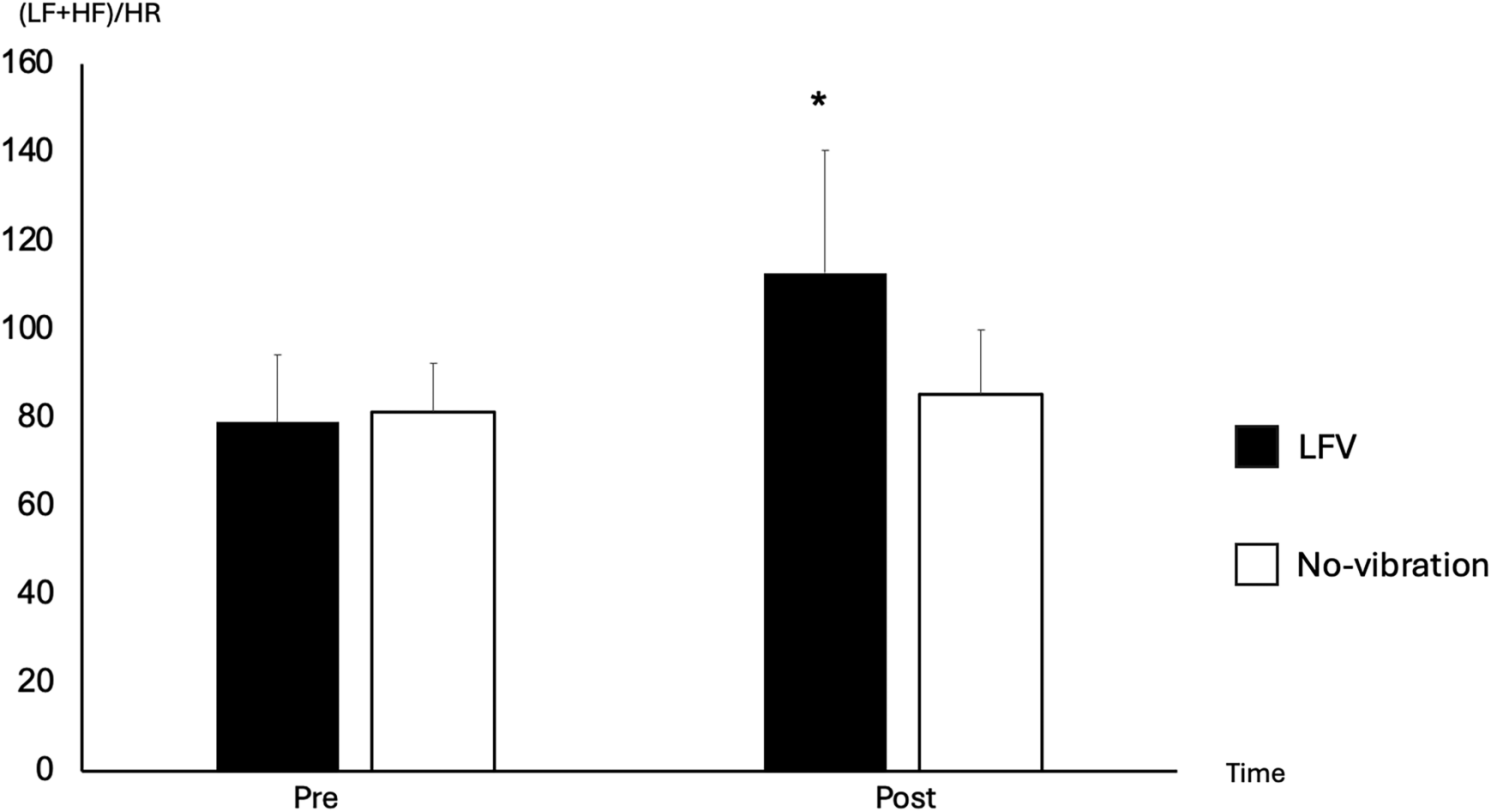
Comparison of (LF + HF)/HR between pre- and post-30 in No-vibration and LFV. Focus on the differences of (LF + HF)/HR between pre- and post 30 in LFV (black) and No-vibration (white), *p < 0.05 significant differences with pre-. We can see a 166% increase in (LF + HF)/HR between pre- and post-30 in the LFV session vs. 121% in the No-vibration session. A strong main effect of SESSION was observed [*F*_(1,130)_ = 27.24, *p* < .0001, *η*^2^ ≈ 0.17], while the TIME main effect was not significant [*F*_(2,130)_ = 1.67, *p* = .1917]. However, the SESSION × TIME interaction was significant [*F*_(2,130)_ = 19.66, *p* < .0001, *η*^2^ ≈ 0.13], suggesting a treatment-specific effect in the modulation of the (LF + HF)/HR ratio.

A significant time effect between pre- and post-30 was observed for HR (*p*-value 0.003), which decreased, and (LF + HF)/HR which increased in the LFV session only (Figure 4) (*p*-value 0.035). No significant difference was observed in RMSSD between pre- and post-30 in both sessions (*p*-value >0.05).

### 3.4 Recovery HRV

The recovery HRVs (comparison between during-20 and post-30) were significant in LFV for (LF + HF)/HR (*p*-value 0.007) and lnRMSSD (*p*-value 0.015). No significant difference was seen between during-20 and post-30 in all HRV variables and HR for the No-vibration sessions (Figure 3).

### 3.5 Comparison between both sessions in post-30 values

Post 30 min HR and HRV variables were not significantly different in both sessions (*p*-value >0.05).

## 4 Discussion

This study aimed to investigate how HRV was modulated during and after a LFV session compared to a No-vibration session in healthy male participants. Consistent with the literature on whole-body vibrations (39, 40), our findings revealed a significant increase in some parasympathetic nervous system metrics 30 min after the LFV session.

It is well known that other interventions such as mindfulness and short sleep can positively influence HRV variables (41–43). Both sessions created relaxing environment, as participants were free from distractions during the intervention. We observed HR fluctuations during the No-vibration and the LFV session with a significant decrease between baseline and during the intervention, showing an increase in parasympathetic tone in both sessions, that could be attributed to the environment of the experiment.

We found significant reactivity HRVs (changes in HRV variables between pre- and during-20) only for the LFV session in lnRMSSD, while the No-vibration session showed no difference in HRV. Additionally, a significant difference was found at during-20 between both sessions with a lower lnRMSSD in LFV. LFV therefore seems to create an acute stress reaction on the body of the participants, showing a decrease in HRV variables during the session. It has been previously demonstrated that chronic exposure to high dose of low-frequency noise during a lifetime can induce vibroacoustic disease, showcasing the stressor effect of such frequencies on the body (44, 45).

In contrast, recovery HRVs (comparison between during-20 and post-30) were found to be statistically different in two variables (lnRMSSD and LF + HF/HR) for the LFV session only. As this increase of HRV variables was also coupled to a decrease in HR, we can assume an enhanced parasympathetic tone thus favoring recovery capacity of the body after a LFV session in comparison to the No-vibration session. When analyzing the reactivity and recovery HRV metrics during the LFV session, we observe a highly dynamic pattern, indicating a significant physiological and psychological response to the vibrations. In contrast, the No-vibration session demonstrates relatively stable HRV metrics, reflecting minimal changes in reactivity and recovery. The effect of a controlled acute stressor on the body could therefore lead to an enhanced recovery reaction of the body.

Comparing HRV variables between baseline (pre-session) and after the intervention (post-30), we found no significant difference in all HRV variables and HR in the No-vibration session between pre- and post-30 measures. In contrast, LFV is showing a relative boost (140.68%) in vagally-mediated HRV variables at 30 min post-session in (LF + HF)/HR coupled by a decrease of HR. A better understanding of the physiological process of LFV would help understanding how the recovery could be affected by this enhancement of HRV variables at 30 min post LFV.

Protocols of other studies investigating the link between LFV and HRV being widely unhomogenized especially in the type of vibrations and length of exposition, a direct comparison of our results is not possible. Most of the literature is based on shorter LFV sessions or is analyzing long-term effect of professional exposure to LFV (5–7).

Despite variations in exposure frequencies, LFV has consistently been shown to influence HRV. LFV has been shown to modulate the ANS through various physiological mechanisms, primarily via mechanotransduction and neural stimulation. Mechanotransduction, the process by which mechanical stimuli like vibration are converted into biochemical signals, occurs through specialized mechanoreceptors such as Pacinian corpuscles and Ruffini endings (46). These receptors transmit afferent input to the central nervous system, where some signals—particularly those originating from visceral or cervical regions—may engage vagal afferents, contributing to increased vagal tone and parasympathetic activation (47–49).

Additionally, vibration therapy has been linked to pain relief, supported by the Gate Control Theory of Pain, where mechanoreceptive input overrides nociceptive signals, thus reducing pain and indirectly modulating the ANS by decreasing stress (50, 51). Low-frequency vibrations influence central nervous system pathways in the brainstem, creating a positive feedback loop, leading to greater parasympathetic activation and a relaxation response (52–54). Finally, vibration affects muscle spindles and Golgi tendon organs, proprioceptive receptors that play a key role in muscle tension and tone regulation, modulating both muscular and autonomic responses (46, 55). The modulation of blood flow, facilitated by vibration-induced muscle stimulation and enhanced circulation, may further promote parasympathetic dominance, influencing blood pressure regulation and overall autonomic tone (56, 57). Collectively, these mechanisms suggest that LFV can be a valuable tool for enhancing autonomic regulation.

Our study offers a new perspective on exposure to LFV on HRV. It is important to note that AudioVitality's technology is unique and therefore making a direct comparison with previous studies impossible. Nevertheless, our study results are going in the same direction as previous literature, showing a positive effect of LFV on HRV mediated variables and ANS stimulation. Our study-design additionally offers an intra-subjects' comparison which limit the high inter-individual's differences in HRV. Further studies with a similar technology could confirm the validity of these results and put into perspectives the validity of our results.

### 4.1 Limitations and recommendations

In our study, HRV measurements were limited to 30 min post-intervention, raising questions about the duration of the post-treatment effects. It remains unclear whether the observed effects persist for a significant period, warranting further research into the chronic impact of LFV. Additionally, our study examined the effects of a single LFV session on HRV, while most AudioVitality protocols involve multiple sessions. Despite the short duration of our protocol, the results offer promising insights into the acute effects of LFV and highlight the need to explore its potential long-term benefits.

The study group consisted of healthy participants with no major symptoms of fatigue nor pain. The extrapolation of the results to other populations will need to be further assessed in future studies.

The study's design was constrained by the available technology, as both LFV and No-vibration sessions needed to occur in the same specialized environment. The lack of randomization possible with such technology created an inherent limitation to our study reducing internal validity. Another potential limitation of within-subject designs is participant habituation to experimental conditions (17). Nonetheless, our within-subject comparison helps mitigate high inter-individual variability, strengthening the reliability of the results despite these constraints.

We implemented strict standardization in measures, including pre-test instructions and consistent measurement protocols. However, some variations were still observed, such as a higher resting heart rate in the LFV session. This suggests that stricter control of external factors affecting HRV could be beneficial to reduce variability and enhance the reliability of future studies on LFV.

## 5 Conclusion

This study aimed to assess the impact of LFV on the ANS by measuring heart rate variability in a healthy active male cohort. Our findings demonstrated a significant increase in some vagal tone markers post-intervention in the LFV session compared to the No-vibration session. Although our technology differs from those commonly cited in the literature, the results align with other. Enhancing vagal tone through LFV could introduce a novel approach to recovery technologies, with potential applications extending to stress management. Further research on LFV is needed to validate these findings and explore broader applications.

## Data Availability

The original contributions presented in the study are included in the article, further inquiries can be directed to the corresponding author.
